# Decomposing socioeconomic inequality in household out of pocket health expenditures in Pakistan (2010-11–2018-19)

**DOI:** 10.1186/s12913-024-11203-9

**Published:** 2024-07-24

**Authors:** Muhammad Zubair, Lubna Naz, Shyamkumar Sriram

**Affiliations:** 1 Collage of Economics and Social Development, Institute of Business Management(IoBM), Karachi, 75190 Pakistan; 2 School of Economics and Social Sciences, Institute of Business Administration (IBA), Karachi, 75270 Pakistan; 3 College of Health Sciences and Professions, Ohio University, Athens, OH 45701 USA

**Keywords:** Socioeconomic inequality, Catastrophic health expenditure, Concentration index, Oaxaca-Blinder decomposition

## Abstract

**Background:**

The increased socioeconomic inequality in catastrophic health expenditure (CHE) disproportionately affects disadvantaged populations, subjecting them to financial hardships, limiting their access to healthcare, and exacerbating their vulnerability to morbidity.

**Objectives:**

This study examines changes in socioeconomic inequality related to CHE and analyzes the contributing factors responsible for these changes in Pakistan between 2010-11 and 2018-19.

**Methods:**

This paper extracted the data on out-of-pocket health expenditures from the National Health Accounts for 2009-10 and 2017-18. Sociodemographic information was gathered from the Household Integrated Economic Surveys of 2010-11 and 2018-19. CHE was calculated using budget share and the ability-to-pay approaches. To assess socioeconomic inequality in CHE in 2010-11 and 2018-19, both generalized and standard concentration indices were used, and Wagstaff inequality decomposition analysis was employed to explore the causes of socioeconomic inequality in each year. Further, an Oaxaca-type decomposition was applied to assess changes in socioeconomic inequality in CHE over time.

**Results:**

The concentration index reveals that socioeconomic inequality in CHE decreased in 2018-19 compared to 2010-11 in Pakistan. Despite the reduction in inequality, CHE was concentrated among the poor in Pakistan in 2010-11 and 2018-19. The inequality decomposition analysis revealed that wealth status was the main cause of inequality in CHE over time. The upper wealth quantiles indicated a positive contribution, whereas lower quantiles showed a negative contribution to inequality in CHE. Furthermore, urban residence contributed to pro-rich inequality, whereas employed household heads, private healthcare provider, and inpatient healthcare utilization contributed to pro-poor inequality. A noticeable decline in socioeconomic inequality in CHE was observed between 2010 and 2018. However, inequality remained predominantly concentrated among the lower socio-economic strata.

**Conclusion:**

These results underscore the need to improve the outreach of subsidized healthcare and expand social safety nets.

## Introduction

Following the United Nations Sustainable Development Framework 2016–2023, developing countries have undertaken concrete steps toward providing universal health coverage (UHC) to meet the healthcare needs of all people, regardless of their ability to pay [1, 2]. The evidence shows that the financial hardship is a stumbling block to equitable access to healthcare in developing countries, particularly with a larger share of the out-of-pocket (OOP) healthcare payment [3]. The OOP medical expenses refer to payments incurred to buy healthcare services directly by the household. The share of OOP health payments is 41% in low-income countries compared with 22% in high-income countries. For Low Middle Income and Upper Middle Income, OOP health expenditure constitute 40% and 31% of total healthcare spending, respectively [4] indicating that low-income countries mostly rely on private healthcare financing, especially OOP health expenditure.

In Pakistan, a significant proportion of healthcare cost is borne by the private sector because of the limited provision of healthcare in the public sector. According to healthcare financing data for FY 2019–2020, approximately 40%, 59.5%, and 0.5% of healthcare is funded by the general government, private sector, and development and donor agencies, respectively. The expenses incurred by the private sector, a substantial 89%, are categorized as OOP payments, which are directly borne by individuals [5]. The reliance on OOP expenses leaves individuals inadequately safeguarded against financial difficulties, often resulting in unmet healthcare needs. Moreover, financial hardships can exacerbate socioeconomic inequalities in health, leading to a decline in overall health status [6]. The expenditure on health is said to be catastrophic when OOP payments for health exceed a 10% threshold of total household expenditure or 40% of household non-food expenses [7].

Catastrophic health expenditure (CHE) can drive households toward impoverishment [8, 9]. Dependence on OOP healthcare financing puts households at risk of decreased accumulated savings or borrowing. When healthcare costs outweigh a household’s financial resources, a substantial disruption in the standard of living occurs, potentially reaching a catastrophic proportion [10]. CHE exacerbates socioeconomic inequality in healthcare; for instance, in Brazil, during 2002–2009, socioeconomic disparities in CHE increased considerably, reaching 5.2 times higher for the poorest individuals and 4 times higher for those with the least education [11]. Similarly, poor households residing in both slum and non-slum areas of Hanoi, Vietnam, face a heightened risk of CHEs. However, only poor households in slums are at a higher risk of falling into impoverishment because of healthcare expenses [12]. Over the past decade, India’s average OOP health expenditure has shown an increasing trend. Wealthier households tend to allocate a considerable proportion of their spending to inpatient care, whereas poorer households tend to spend more on outpatient care [13].

Few studies have examined the incidence and factors contributing to CHE in Pakistan [14, 15]. Consequently, a significant gap exists in the literature on the analysis of socioeconomic inequality in relation to CHE in Pakistan. This study fills this gap by examining the socioeconomic inequality in CHE in Pakistan. First, it examines whether the CHE incidence has changed over time (2010–2018), aiming to determine whether the incidence has increased or decreased? Second, it investigates the socioeconomic inequality in CHE, with the objective of assessing the existence and magnitude of CHE inequality in Pakistan. Furthermore, it explores the factors contributing to socioeconomic inequality in CHE and the extent to which they contribute. Finally, it assesses changes in socioeconomic inequality over time, aiming to analyze whether such inequality is static or persists over time.

## Methodology

### Data and sample

The study obtained data from the two rounds of Household Integrated Economic Surveys (HIES) in 2010-11 and 2018-19 and National Health Accounts of 2009-10 and 2017-18. Pakistan Bureau of Statistics conducts National Health survey prior to the HIES in alternate years. The household survey (called as HIES) extensively cover various socioeconomic indicators, including health, education, housing, population welfare, water sanitation and hygiene, income and expenditure, information communication and technology, and the Food Insecurity Experience Scale. The Pakistan Bureau of Statistics employed different approaches for data collection before and after 2017. The sampling was based on the 1998 census before 2017, whereas the 2018–2019 sample was constructed using data from the 2017 census. To ensure comprehensive coverage, enumeration blocks were selected for the survey in all the four provinces (Punjab, Sindh, Khyber Pakhtoonkhwa, and Balochistan). On average, each enumeration block consisted of 200–250 houses. The enumeration blocks were further categorized into rural and urban areas. These enumeration blocks were treated as primary sampling units in urban regions, whereas villages were divided into blocks with clearly defined boundaries and maps in rural areas. This study extracted a sample of 5,126 households from 2010-11 and 24,809 households from 2018-19.

### Catastrophic health expenditures

There are two widely accepted approaches to measure CHE. The budget share method examines CHE if OOP healthcare expenditure exceeds 10% of a household’s total consumption expenditure [10], while the capacity-to-pay (CTP) method analyzes OOP that surpasses 40% of a household’s total consumption after subtracting basic subsistence needs such as housing and food expenses.

1 $${h}_{i}=0\;if\left\{\frac{OO{P}_{i}}{{E}_{i}-f\left({E}_{i}\right)}\right\}\le {\rm Z}$$

$${h}_{i}=1\;if\left\{\frac{OO{P}_{i}}{{E}_{i}-f\left({E}_{i}\right)}\right\}\le {\rm Z}$$

${h}_{i}$ = (Catastrophic Healthcare expenditure (CHE) / Total expenditures of Household)

$OOP_{i}$ = Out of pocket healthcare expenditures

${E}_{i}$= Total expenditures of Household

$f({E}_{i})$= Household expenditures on food = Given CHE threshold

The budget share method tends to underestimate CHE for poorer households but overestimates it for wealthier households. For low and middle-income groups, the CTP approach is more appropriate [7]. Several studies have used CTP to assess socioeconomic inequality in CHE [6, 16–18]. However, we used both on budget share and CTP approaches to measure socioeconomic inequality in CHE.

#### Concentration index

The concentration index (CI) is commonly used to measure socioeconomic inequality in CHE. In the assessment of inequality concerning CHEs, it is appropriate to employ the standard concentration index (SCI) for relative inequalities and generalized concentration index (GCI) for absolute inequalities. The Erreygers index is best suited for bounded variables but may not be appropriate for continuous variables [19]. In this study, we used both relative and absolute measures to assess socioeconomic inequality in CHE. We used the relative concentration index (RCI) and concentration curve (CC) (which plots the cumulative proportion of CHE on the vertical axis against the cumulative share of the population ranked by socioeconomic status e.g., household expenditure on the horizontal axis). When everyone experiences a similar CHE burden, the curve is a 45-degree line, indicating “perfect equality.” RCI twice the area between the CC and 45-degree line. If the CC is above the 45-degree line, the SCI value is negative, indicating that the burden of CHE is higher among low socioeconomic groups (poor). If it is below the line, the SCI value is positive, indicating a higher burden among high socioeconomic groups (rich). The SCI value ranges from − 1 to + 1, with 0 representing “perfect equality” [6, 20].

2 $${C}\left({h}\right|{y})=\frac{2{cov}({{h}}_{{i}},{{R}}_{{i}})}{\overline{{h}}}=\frac{1}{{n}}{\sum }_{{i}=1}^{{n}}\left[\frac{{{h}}_{{i}}}{{h}}\left(2{{R}}_{{i}}-1\right)\right]$$

Where, n = The total number of the samples.

h_i_ = Either the household incurs CHE or not.

R_i_ – 1, = The fractional range of per capita expenditures, with i = 1 for poorest and i = n for the richest individuals.

The generalized concentration curve (GCC) represents the cumulative share of the population, ordered by ascending socioeconomic status (SES), and cumulative proportion of CHE multiplied by the parameter µ (the mean of CHE). The GCI is computed as double the area between the GCC and line of perfect equality, obtained by multiplying the RCI with µ [21]. The Generalized Concentration Index (GCI) can be expressed as

3 $${G}{C}{I}\hspace{0.17em}=\hspace{0.17em}{C}\left({h}\right|{y})=\frac{1}{{n}}{\sum }_{{i}=1}^{{n}}\left[{{h}}_{{i}}\left(2{{R}}_{{i}}-1)\right)\right]$$

The SCI fails to capture the “mirror” properties. For a given health distribution, for instance, the health index I(h) and corresponding ill health index I(s) have equal absolute values but opposite signs [22]. Therefore, we used the Erreygers index, considering the precedence of the mirror condition. This index can be expressed as

4 $${E}\left({a}\right|{y})= \frac{1}{{n}}{\sum }_{{i}=1}^{{n}}\left[\frac{4{{a}}_{{i}}}{\left(\right.{{a}}^{{m}{a}{z}-}{{a}}^{{m}{i}{n}\left.\right)}\left(2{{R}}_{{i}}-1\right)}\right]= -{E}({s}\left|{y}\right)$$

This index ranges between −1 and + 1.

#### Decomposition of concentration index

The Wagstaff, Adam, Van Doorslaer, and Watanabe method for decomposition of socio-economic inequality has been used widely in literature [6, 16, 18]. Due to its comprehensiveness and wide applicability [23, 24] this study also uses this method to decompose socioeconomic inequality in CHE. It decomposes the concentration index of relative OOP health expenditure into its socio-economic ingredients [7]. A decomposition analysis was conducted by examining the CI for the dependent variable CHE(*y)*, followed by each contributing factor $(\chi )$. Subsequently, a linear regression model was used to determine the absolute contribution of each factor ($\chi$) to the CI of CHE.

5 $${y}={\alpha }+{\sum }_{{\kappa }}{{\beta }}_{{\kappa }}{{\chi }}_{{\kappa }}+{\epsilon }$$

Given the relationship between dependent explanatory variables CI for *y is written as*

6 $${C}{I}=\sum \left(\frac{{{\beta }}_{{\kappa }}{{\chi }}_{{\kappa }}}{{\mu }}\right){C}{{C}}_{{\kappa }}+\frac{{G}{{C}}_{{\epsilon }}}{{\mu }}$$

Where $\text{X}_{\kappa}$ = mean of covariates

µ = the mean of the binary health outcome, specifically households with CHE.

$\text{CCI}_{\kappa}$ = concentration index of the determinants, calculated using Eq. 2.

k = the marginal effect of parameter $\beta_{\kappa}$, and GCε/µ denotes the unexplained inequality in CHE.

A negative CI suggests a pro-poor distribution of the variable, whereas a positive CI indicates a pro-rich distribution. The absolute contribution quantifies each explanatory variable’s contribution to socioeconomic inequality in CHE. A positive absolute contribution implies a preference for worse-offs, whereas a negative absolute contribution implies a preference for better-offs.

### Decomposition of changes in concentration index

The Oaxaca–Blinder decomposition approach [25, 26] allows changes in outcomes between the two groups that can be decomposed by various factors. One is the difference in the distribution or levels of characteristics within these groups, known as the distributional effect. The other part is due to differences in how these characteristics affect the outcomes within each group, termed the coefficient effect. Decomposition analysis examines the relationship between an outcome variable and a set of observed characteristics. Wagstaff et al. [7] introduced an Oaxaca-type decomposition of inequality to examine the impact of temporal changes on the determinants of health inequality. We used this approach to assess temporal changes in the decomposition of inequality in CHE between 2010-11 and 2018-19 in Pakistan.

7 $${\varDelta }{C}={\sum }_{{\kappa }}{{\eta }}_{{\kappa }{\tau }}\left({{C}}_{{\kappa }{\tau }}-{{C}}_{{\kappa }{\tau }-1}\right)+{\sum }_{{\kappa }}{{C}}_{{\kappa }{\tau }-1}\left({{\eta }}_{{\kappa }{\tau }}-{{\eta }}_{{\kappa }{\tau }-1}\right)+{\varDelta }\left(\frac{{G}{{C}}_{{\epsilon }{\tau }}}{{{\mu }}_{{\tau }}}\right)$$

The difference in concentration index ${{C}}_{{\kappa }{\tau }}-{{C}}_{{\kappa }{\tau }-1}$ is weighted by the second period’s (2018) elasticity $\eta_{\kappa \tau }$, while the difference in elasticity $\eta_{\kappa \tau }-\eta_{\kappa \tau -1}$ is weighted by the first period’s (2010) concentration index $C_{\kappa \tau -1}$ [16, 20].

## Results

### Measurement of catastrophic health expenditures

CHE was measured using both the budget approach (10% of total expenditure) and CTP method (40% of non-food expenditure) for the years 2010-11 and 2018-19. To ensure the robustness of the results, other cutoffs, including 15% and 20% of total expenditure and 20% of non-food expenditure, were also utilized. Using the budget approach, the results show that 5.6% of the population in 2010 and 13.3% of the population in 2018 experienced CHE (Table 1). CTP reveals that 6.9% and 3.9% of the population faced CHE in 2010-11 and 2018-19, respectively. A comparison of CHE in 2010-11 and 2018-19 indicates that CHE expenditure decreased in 2018-19 when the CTP approach was used, whereas CHE increased when the total expenditure approach was employed. The other cutoffs used to calculate CHE are reported in Table 1 show a similar trend over time.

**Table 1:** Catastrophic health expenditure 2010-11 and 2018-19 in Pakistan at various cut-offs used

Cutoff	2010-11	2018-19
@10% of total expenditures	5.6%	13.3%
@15% of total expenditures	3.3%	7.1%
@20% of total expenditures	2.1%	4.4%
@20% of non-food expenditures	13.7%	12.0%
@40% of non-food expenditures	6.7%	3.9%

Table 2 presents the socioeconomic characteristics of households that experienced CHE in 2010-11 and 2018-19. In 2009–10, 70% of the households that experienced CHE lived in rural areas. This number increased to 74% when the CTP approach is applied but decreased to 67% when the budget share approach is used in 2018–19. The prevalence of CHE in male-headed households is higher than that in other groups with similar characteristics.

**Table 2:** Socio-economic characteristics of households experiencing catastrophic health expenditures

Variable	Category	2010-11		2018-19	
		At 10% of budget share	AT 40% CTP	At 10% of budget share	AT 40% CTP
Place of residence	Rural	70.17	70.86	67.42	74.81
	Urban	29.83	29.14	32.58	25.19
Gender of household head	Male	84.91	85.92	89.96	90.43
	Female	15.09	14.08	10.04	9.57
Age of household head	16–30 years	18.87	18.31	13.78	13.70
	31–50 years	39.62	43.66	45.78	42.02
	51–60 years	30.19	25.35	19.74	19.15
	60 years and above	11.32	12.68	20.71	25.13
Head’s education	Attended school	80.68	79.14	45.68	41.71
	Never attended school	19.32	20.86	54.32	58.29
Head’s employment	Employed	37.97	34.86	44.46	43.96
	Unemployed	62.03	65.14	55.54	56.04
Type of healthcare	Outpatient	77.63	80.29	85.31	78.59
	Inpatient	21.69	19.14	9.77	15.59
	Self-medication	0.68	0.57	4.91	5.82
Type of healthcare provider	Private	85.08	86.86	80.52	80.41
	Public	14.92	13.14	19.48	19.59
Wealth status	Poorest	45.76	44.57	26.48	30.13
	Poorer	23.73	23.71	22.40	21.62
	Middle	18.98	18.57	18.68	17.73
	Richer	7.80	8.57	18.15	17.56
	Richest	3.73	4.57	14.28	12.95
Household size	1–3	12.88	13.43	29.86	31.50
	4–6	40.00	38.29	40.54	38.14
	7	47.12	48.29	29.60	30.35

CHE is higher in 2018-19 compared to 2010-11. Furthermore, households with uneducated heads experienced CHE more than those with educated ones in 2010-11 than in 2018-19. Similarly, in 2010-11, smaller households have a greater proportion of CHE incidence than larger households. However, during 2018-19, smaller households have a smaller share of CHE incidence. The results show a decrease in the percentage of households with the lowest SES (poorest) and an increase in the percentage of households with the highest SES (richest) in 2018-19 compared with 2010-11. For instance, the richest quintile has 4.6% CHE for 2010-11 (CTP approach), which increased to 13% for 2018-19. Similarly, the poorest quintile is 45.7%, which is reduced to 30% for 2018-19.

The logistic regression was applied to investigate the factors linked to CHE in both 2010-11 and 2018-19, and results are presented in Table A2 and A3 in the appendix. The findings indicate that determinants of CHE, including household wealth status, education level, and private healthcare provider, remain consistent across the two time periods. However, a greater number of statistically significant factors were identified in 2018-19 compared to 2010-11. In 2010-11, the odds of incurring CHE lowered for the richest quantile but increased for private healthcare provider. In 2018-19, the odds of incurring CHE decreased for the richest quantile, household’s heads aged between 31 and 50 years, employed heads of households, and medium-sized households. However, the odds of incurring CHE, for households with heads aged over 60 years, urban region, and private healthcare providers increased.

### Socioeconomic inequality in catastrophic health expenditures

**Fig. 1:**
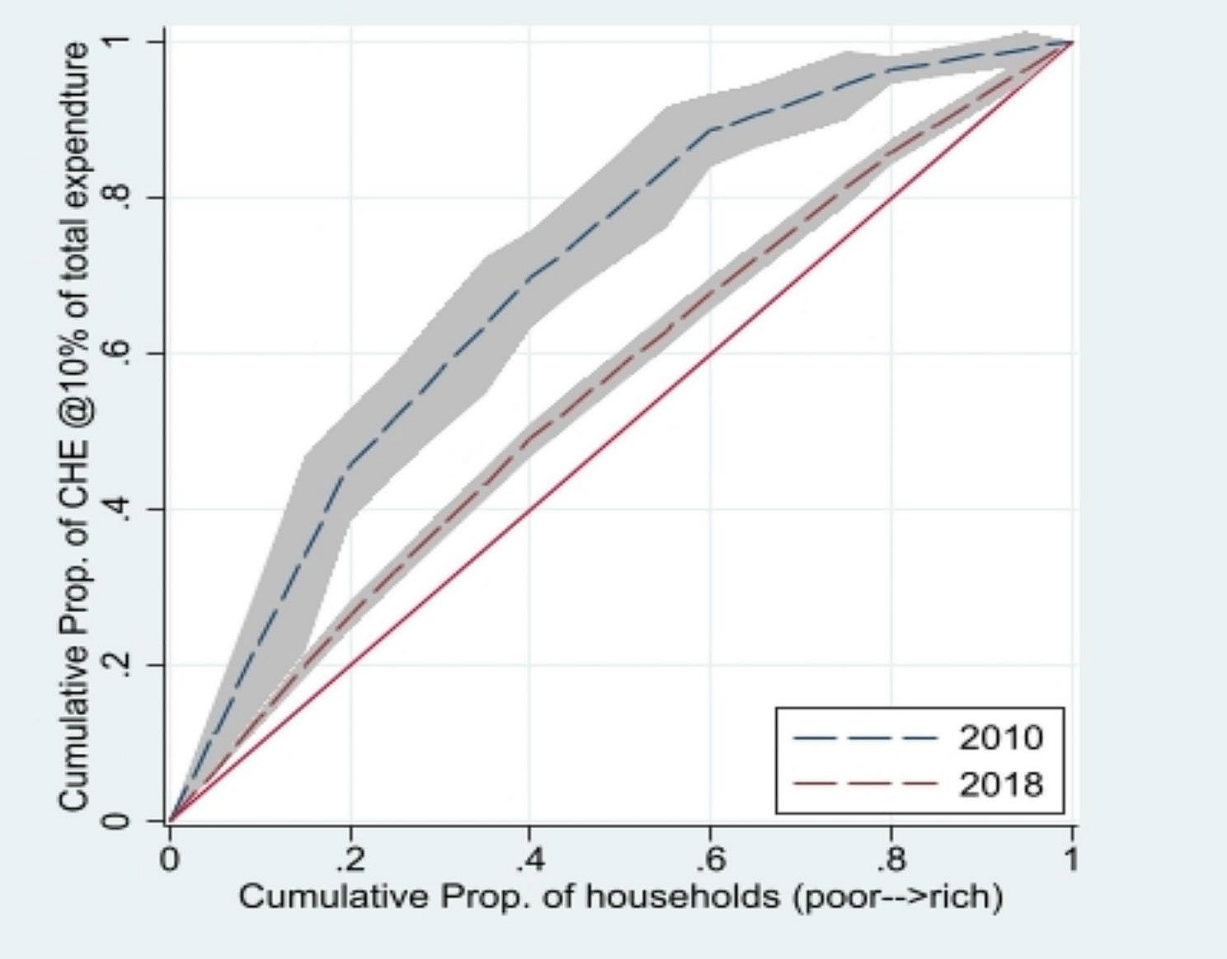
Concentration Curve (CHE at 10% of total expenditure)

**Fig. 2:**
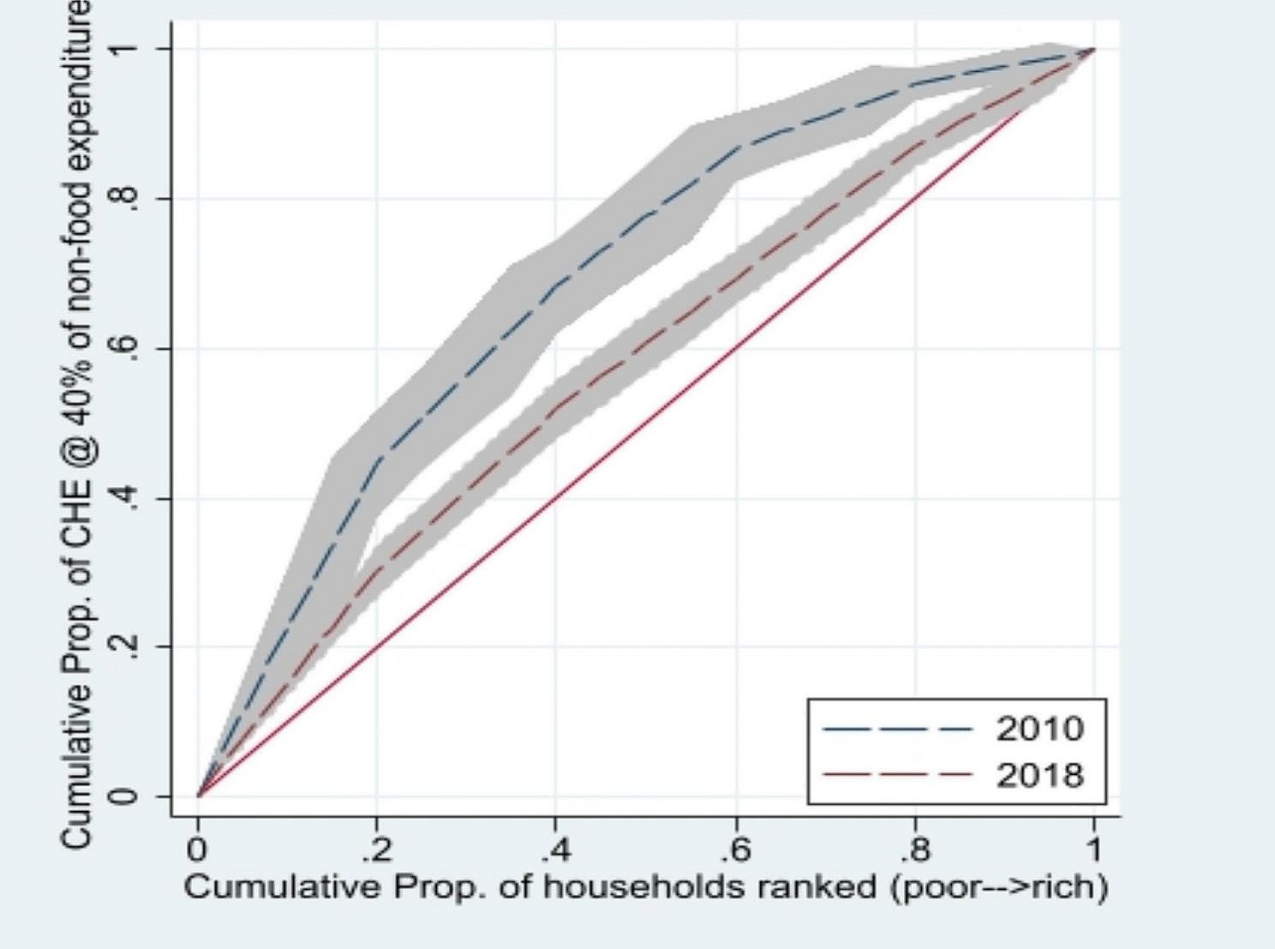
Concentration Curve (CHE at 40% of non-food expenditure)

Table 3 presents the results of the CIs (standard, generalized, and Errygers) calculated using budget share and CTP approaches for the years 2010-11 and 2018-19. The results reveal that CHE is concentrated in the low socioeconomic status group (poor), as indicated by the negative values of the CI. Additionally, Fig. 1 shows that the CC lies above the line of equality for 2010-11 and 2018, indicating greater variation in CHE among poor households than that among rich households. Comparing the results of the standard, generalized, and Erryegers indices (Table 3), the values of all CIs decreased in 2018-19 compared to  2010-11. Moreover, Fig. 1 shows that the CC for 2018-19 lies below the CC of 2010-11, implying that socioeconomic inequality reduced in 2018-19 compared with 2010-11. Table A1 in the appendix presents the findings of urban vs. rural socioeconomic inequality in CHE. The results for Standard, Generalized, and Errygers Indices indicate that CHE is predominantly concentrated among the poor in both rural and urban areas for both 2010-11 and 2018-19. Despite a reduction in values, socioeconomic inequality was higher in rural areas than in urban areas in 2010-11. However, the results were reversed in 2018, as the concentration index shows that Urban socioeconomic inequality in CHE outstrip that in rural areas. Moreover, the reduction in the concentration of socioeconomic inequality among the poor was noticed in 2018 at the national level, (See Fig. 2).

**Table 3:** Concentration indices in 2010-11 and 2018-19

Year	Index value@10% of monthly total expenditures			*P*-value	Index value @40% of monthly non-food expenditures			*P*-value
	Standard	Generalized	Erreygers		Standard	Generalized	Erreygers	
2010-11	− 0.399	-0.023	-0.092	< 0.001	-0.385	-0.025	-0.103	< 0.001
2018-19	-0.114	-0.015	-0.063	< 0.001	-0.153	-0.006	-0.027	< 0.001
Difference	-0.285	-0.008	-0.028		-0.22	-0.019	-0.076	

### Decomposition of socioeconomic inequality

Tables 4 and 5 present the results of the Wagstaff decomposition of inequality in CHE for 2010 and 2018, respectively. Table 4 shows the results of the decomposition of inequality in CHE measured by the budget share approach, whereas Table 5 shows the decomposition results in CHE calculated using the CTP pay approach for 2010-11 and 2018-19.

**Table 4:** Decomposition of catastrophic health expenditures (CHE at 10% of total expenditures) in 2010-11 and 2018-19

Variables	2010-11				2018-19			
	Elasticity	CI	Contribution		Elasticity	CI	Contribution	
			Absolute	Percentage			Absolute	Percentage
**Wealth status (Ref. Poorest)**								
Poorer	-0.019	-0.079	0.005	-23.0	-0.020	-0.079	0.002	-14.6
Middle	-0.022	0.000	-0.000	0.01	-0.039	0.000	-0.000	0.00
Richer	-0.030	0.080	-0.008	36.4	-0.045	0.080	-0.005	33.0
Richest	-0.035	0.159	-0.019	84.7	-0.068	0.159	-0.015	99.5
**Gender of household head (Ref. Male)**								
Female	0.022	-0.001	-0.00001	0.06	-0.034	-0.006	0.00006	-0.40
**Age of household head (Ref. 16-30 years)**								
31–50 years	-0.005	-0.001	0.00001	-0.04	-0.010	-0.021	0.0003	-2.41
51–60 years	0.027	-0.007	-0.00001	0.06	-0.009	0.011	-0.000	0.47
Above 60 years	-0.025	0.001	-0.00002	0.09	0.042	0.009	0.0002	-1.37
**Head’s education (Ref. Never attended school)**								
Attended school	0.0006	0.009	0.000	-0.38	0.001	0.067	0.0003	-1.89
**Head’s employment (Ref. Unemployed)**								
Employed	0.0002	0.0013	0.000	-0.00	-0.009	-0.035	0.001	-8.18
**Household size (Ref. 1–3)**								
4–6	0.0008	-0.011	0.0003	-0.14	-0.004	-0.009	0.0001	-0.92
7	0.0001	0.011	-0.001	0.06	0.001	0.095	0.0002	-1.39
**Place of residence (Ref. Rural)**								
Urban	-0.001	0.011	-0.0001	0.57	-0.002	0.078	-0.0004	2.68
**Type of healthcare provider (Ref. Public)**								
Private	0.002	-0.001	0.00006	-0.29	0.005	0.007	0.0002	-1.54
**Type of healthcare (Ref. Outpatient)**								
Inpatient	0.702	0.002	-0.0004	1.88	0.898	0.001	0.0004	-2.96
Residual			-2.082	9.0			-0.00006	3.40
Total			-0.023	100			-0.015	100

**Table 5:** Decomposition of catastrophic health expenditures in 2010-11 and 2018-19 (CHE at 40% of monthly non-food expenditures)

Dependent variable	2010-11				2018-19			
	Elasticity	CI	Contribution		Elasticity	CI	Contribution	
			Absolute	Percentage			Absolute	Percentage
**Wealth status (Ref. Poorest)**								
Poorer	-0.025	-0.079	0.005	-23.0	-0.003	-0.079	0.001	-20.4
Middle	-0.030	-0.000	-0.000	0.01	-0.005	0.000	-0.000	0.00
Richer	-0.040	0.080	-0.009	36.3	-0.005	0.080	-0.002	31.4
Richest	-0.046	0.159	-0.021	84.5	-0.008	0.159	-0.006	88.5
**Gender of household head (Ref. Male)**								
Female	0.013	-0.00	-0.000	0.02	-0.008	-0.006	0.0005	-0.76
**Age of household head (Ref. 16-30 years)**								
31–50 years	0.005	-0.001	-0.000	0.03	-0.001	-0.021	0.0001	-2.35
51–60 years	0.036	-0.000	-0.00001	0.06	-0.001	0.011	-0.00002	0.39
Above 60 years	-0.007	0.001	-0.00	0.01	0.009	0.009	0.0001	-2.33
**Head’s education ** **(Ref. Never attended school)**								
Attended school	0.0004	0.009	0.00004	-0.17	-0.0001	0.067	-0.0001	2.00
**Head’s employment (Ref. Unemployed)**								
Employed	-0.001	0.001	-0.000	0.03	2.31	-0.035	0.0004	-6.32
**Household size (Ref. 1–3)**								
4–6	0.0002	0.005	0.000	-0.02	-0.000	-0.009	0.00008	-1.17
7	0.0001	-0.01	-0.000	0.03	-4.440	0.095	-0.00002	0.43
**Place of residence (Ref. Rural)**								
Urban	-0.002	0.01	-0.0001	0.63	-0.001	0.078	-0.001	16.4
**Type of healthcare provider (Ref. Public)**								
Private	0.003	0.002	0.0008	-0.35	0.0007	0.007	0.0001	-1.53
**Type of healthcare (Ref. Outpatient)**								
Inpatient	0.86	-0.001	-0.0004	1.72	0.179	0.001	0.0002	-4.29
Residual			-1.06E	4.0			-0.00004	3.39
Total			-0.025	100			-0006	100

The findings indicate that the primary contributors to inequality in CHE are the wealth quantiles (the second, fourth, and fifth quantiles) in 2010-11. Among the contributors to inequality in CHE, upper wealth quantiles and inpatient healthcare were statistically significant, signifying that these variables favor the better-off and disadvantage the poor. Other factors, including lower wealth quantiles, negatively affect CHE inequality, indicating the opposite.

Similarly, in 2018-19, the primary contributors to CHE inequality are the wealth quantiles, followed by urban residence. Among the statistically significant contributors to inequality in CHE, higher wealth quantiles and urban residence have a positive impact on CHE inequality, indicating that these variables favor the better-off and disadvantaged the poor. Other factors, such as the lower wealth quantiles and employed head of household, are negative contributors to inequality in CHE, meaning that they favor the poor and disadvantage the rich. The unexplained factors for 2010-11 and 2018-19 are positive and significant contributors to inequality in CHE, implying that they favor the better-off and disadvantage the poor. The share of unexplained contributors decreased from 9 to 4% in the inequality of CHE calculated using the total expenditure approach; however, it remained unchanged when the non-food expenditure approach was used.

### Changes in inequality of catastrophic health expenditures

Table 6 presents the Oaxaca-type decomposition of changes in inequality. Overall, there is -0.008 (53.4%) reduction in inequality in CHE when calculated using the total expenditure approach. In contrast, there is a more substantial decrease of -0.019 (316%) in CHE inequality when computed using non-food expenditure. The observed socioeconomic characteristics explain 92.7% of the change in inequality in CHE calculated using the total expenditure approach and 98.6% of the change in inequality in CHE computed using non-food expenditure. The remaining portion of CHE inequality is attributable to unobservable characteristics. Among the contributing factors, higher wealth quantiles are the main contributors to changes in inequality in CHE between 2010-11 and 2018-19. They represented a 95.5% increase in inequality in CHE computed by the total expenditure approach and 121.1% in the inequality in CHE computed by the non-food expenditure approach. Contrastingly, the lower wealth quantiles contributed 41.7% and 23.8% to reduce the inequality in CHE computed by the total expenditure and non-food expenditure approaches, respectively. The other contributors that significantly represented the increase in inequality are the employed head of household, inpatient access to healthcare, and private provision of healthcare. In contrast, urban residence contributed to a decline in inequality in CHE between 2010-11 and 2018-19.

**Table 6:** Oaxaca-Blinder decomposition of changes in inequality in catastrophic health expenditure, 2010-11 and 2018-19

Independent variables	CHE at 10% of monthly total expenditure		CHE at 40% of monthly non-food expenditure	
	Change in Absolute contribution	Percentage	Change in Absolute contribution	Percentage
**Wealth status (Ref. Poorest)**				
Poorer	0.002	-41.75	0.004	-23.8
Middle	-2.460	0.03	-3.214	0.01
Richer	-0.003	44.1	-0.007	38.1
Richest	-0.003	51.5	-0.015	83.1
**Gender of household head (Ref. Male)**				
Female	-0.000	1.11	-0.00005	0.31
**Age of household head (Ref. 16-30 years)**				
31–50 years	-0.0003	5.22	-0.0001	0.88
51–60 years	0.000	-0.86	0.000	-0.05
Above 60 years	-0.0002	3.36	-0.000	0.85
**Head’s education (Ref. Never attended school)**				
Attended school	-0.0002	2.99	0.000	-0.94
**Head’s employment (Ref. Unemployed)**				
Employed	-0.001	18.2	-0.0004	2.30
**Household size (Ref. 1–3)**				
4–6	-0.000	1.60	-0.000	0.38
7	-0.0002	3.33	0.000	-0.10
**Place of residence (Ref. Rural)**				
Urban	0.0002	-4.12	0.0009	-4.99
**Type of healthcare provider (Ref. Public)**				
Private	-0.0001	2.47	-0.00001	0.06
**Type of healthcare (Ref. Outpatient)**				
Inpatient	-0.0009	12.6	-0.0007	3.87
Residual	5.19	-7.28	2.213	-1.2E
Total	-0.008	100	-0.019	100

## Discussion

The results suggest that socioeconomic inequality in CHE decreased in Pakistan between 2010-11 and 2018-19. The results are consistent with earlier evidence  [13], which reveal a insignificant reduction in socioeconomic-related inequality in CHE over a similar 10-year span in India. The decrease in CHE inequality in Pakistan can be attributed to increased healthcare outreach, such as the rise in public healthcare spending. General public spending on health in Pakistan has increased substantially [27, 28]. The increase in public sector developmental expenditure expands healthcare resources and improves the health infrastructure, making it convenient for a larger segment of the population to access public health facilities [29], which consequently reduces inequality in CHE. Despite the reduction in CHE inequality, the findings indicate a concentration of CHE within the lower socioeconomic strata (poor). This inference is drawn from the negative values of the CI. The results indicate that individuals with lower socioeconomic status are more susceptible to socioeconomic inequalities in CHE compared with wealthier population in Pakistan in 2010-11 and 2018-19. These findings are in conformity with empirical evidence from other low middle income countries [6, 16, 18]. The healthcare expenditure (CHE) inequality that disproportionately affects the poor in Pakistan is attributed to the reliance on private healthcare financing, which constitutes approximately 60% of healthcare expenditure [5]. Owing to inadequate investment and the resulting gaps in affordable healthcare access, most healthcare expenses are primarily covered through OOP spending, with a substantial portion of healthcare services being offered by private facilities. Consequently, this situation has resulted in a visible inequality in access to healthcare between rich and poor individuals [30]. Further, the findings of this study indicate that the households seeking healthcare privately are more prone to experience Catastrophic Health Expenditure (CHE) compared to those utilizing public sector facilities. Other studies also confirm that use of privately-owned facilities exacerbates socioeconomic inequalities in health [16, 18, 31].

A study from India shows that private healthcare significantly dominates the market, leading to a highly commercialized healthcare sector. Inadequate health insurance support for disadvantaged population leads to the increased CHE inequality [13]. In developing countries, the lower socioeconomic strata allocates a substantial portion of their income toward OOP healthcare expenses, which play a crucial role in perpetuating this pro-poor inequality in CHE because of the unavailability of universal health coverage or UHC [32]. In 2015, Pakistan launched a public health insurance program as part of its UHC initiative, initially implemented in the province of Khyber Pakhtunkhwa [33]. The program was subsequently expanded to other regions of the country, except Sindh. The impacts of this public sector insurance program on inequality in CHE are yet to unfold, as this research only covers the two years after the initiation of the program in Pakistan.

In this study, the contributors to inequality in CHE were primarily wealth quantiles, especially the higher wealth quantiles, which positively contributed to inequality in 2010-11 and 2018-19. In both years, all potential factors except urban residence and higher wealth quantiles contributed to the reduction in CHE inequality in Pakistan. Factors that caused a reduction in CHE inequality were employed head of household and inpatient access to healthcare. Urban residences have a larger positive contribution to inequality in CHE after the wealth quantiles. This indicates rural versus urban socioeconomic inequality, where urban region exhibits lower CHE inequality in 2010-11, but higher CHE inequality in 2018-19. Although healthcare services are often more accessible in urban areas than in rural areas, significant inequalities in the access and utilization of these services persist among various social categories within urban centers [34]. Unobservable factors contributed to a smaller proportion of inequality in CHE, which decreased further in 2018-19 compared to  2010-11. In contrast to previous research [13, 16, 18, 35], which highlighted the significant impact of education on health inequality, the education minimally contributes to CHE inequality in this study. However, as expected, the results suggest that the education negatively contributes to socioeconomic inequality in health which is consistent with the findings of previous studies. Further, the employed head of the household reduced socioeconomic inequality in health, which aligns with previous findings [16]. This can be attributed to the fact that employment leads to an improved economic status and better access to health services which in turn reduces socioeconomic inequality in health.

Overall, despite the reduction, inequality in CHE remains pro-poor, which is a matter of concern in Pakistan. As mentioned above, inequalities in Pakistan extend to sectors other than the healthcare sector. Integrated healthcare policies involving various sectors can help provide much-needed financial risk protection for vulnerable individuals. Pakistan lies behind neighboring countries in terms of good health and well-being indicators. Health outcomes can be improved by strengthening healthcare protection for the poor population [30], which can help mitigate socioeconomic inequalities in healthcare expenditure. Moreover, targeted interventions are required to address inequalities in income and regional disparities to address the exacerbating inequality in health.

## Conclusion

This study analyzes socioeconomic inequality in CHE in Pakistan between 2010-11 and 2018-19. Additionally, it explores the factors contributing to inequality in CHE and the changes in these factors over time. The results revealed a decrease in socioeconomic inequality in Pakistan’s CHE over time. However, inequality remained persistently concentrated in the lower socioeconomic groups of the population. Inequality is primarily driven by wealth status, while other factors, such as household size, household with a female head, employed head of the household, type of healthcare, and healthcare providers, are not significant contributors. Higher wealth quantiles are primarily responsible for changes in CHE inequality in Pakistan. It is important to comprehend the factors that perpetuate this socioeconomic inequality in CHE, as this understanding can help in creating interventions that prioritize the welfare of lower socioeconomic status groups. These interventions can not only alleviate the financial burdens faced by the impoverished due to exorbitant healthcare expenses but also diminish the overarching socioeconomic gaps. Given the persistent pro-poor inequality in CHE over time, the outreach of public health insurance programs, such as the Health card, should be expanded with an equity-based approach. The social safety nets such as the Benazir Income Support Program (BISP) need to be expanded with targeted health subsidies for the poor patients. Additionally, increasing public sector health investment is crucial to achieve Universal Health Coverage (UHC). Further, inter-sectoral coordination which aims to integrate the healthcare policies with poverty reduction strategies can be helpful in addressing the socioeconomic inequality in health.

This study has certain limitations. The use of cross-sectional data constrains our ability to establish a causal relationship between socio-economic inequality in CHE and factors contributing to CHE inequality. Furthermore, the data does not provide insight into how financial status changes over the life cycle of an individuals.  Our results offer a static glimpse into the financial challenges and factors influencing them that they experience at a particular point in time. This study used a Wagstaff-type inequality decomposition, which addresses the extent of variation in health status without explicitly considering the interrelationship between health and socio-economic status or SES [36]. Additionally, this decomposition method is specifically suited for analyzing absolute inequality indices such as the absolute CI, thus restricting its scope [23, 36, 37].

## Appendix

**Table A1:** Concentration Indices (Rural vs. Urban) 2010-11 and 2018-19

Year	Region	Index value@10% of monthly total expenditures			*P*-value	Index value @40% of non-food expenditures			*P*-value
		Standard	Generalized	Erreygers		Standard	Generalized	Erreygers	
**2010-11**	Rural	-0.403	-0.024	-0.099	< 0.001	-0.390	-0.028	-0.115	< 0.001
	Urban	-0.385	-0.019	-0.077	< 0.001	-0.349	-0.020	-0.081	< 0.001
**Difference**		-0.018	-0.005	-0.022		-0.041	-0.008	0.034	
**2018-19**	Rural	-0.073	-0.010	-0.042	< 0.001	-0.088	-0.004	-0.018	< 0.010
	Urban	-0.169	-0.021	-0.084	< 0.001	-0.189	-0.005	-0.023	< 0.008
**Difference**		-0.096	-0.011	-0.042		-0.101	-0.001	-0.005	

**Table A2:** Logistic regression model for determinants of CHE at 10% of household total expenditure

Variable	2010-11		2018-19	
	Odd Ratio	95% Conf. Interval	Odd Ratio	95% Conf. Interval
**Wealth status (Ref. Poorest)**				
Poorer	0.420^***^	0.303 0.582	0.805^***^	0.739 0.878
Middle	0.341^***^	0.241 0.482	0.633^***^	0.576 0.695
Richer	0.139^***^	0.087 0.223	0.588^***^	0.532 0.649
Richest	0.054^***^	0.028 0.103	0.422^***^	0.378 0.472
**Gender of household head (Ref. Male)**				
Female	1.239	0.549 2.793	0.928	0.797 1.081
**Age of household head (Ref. 16-30 years)**				
31–50 years	0.810	0.487 1.346	0.842^***^	0.772 0.919
51–60 years	1.253	0.688 2.285	0.928	0.831 1.037
60 above	0.681	0.284 1.629	1.203^***^	1.082 1.338
**Head’s education (Ref. Never attended school)**				
Attended school	1.237	0.899 1.701	1.036	0.974 1.101
**Head’s employment (Ref. Unemployed)**				
Employed	1.041	0.802 1.350	0.736^***^	0.687 0.789
**Household size (Ref. 1–3)**				
4–6	1.138	0.763 1.697	0.884^***^	0.821 0.952
7	1.010	0.681 1.499	1.037	0.950 1.132
**Place of residence (Ref. Rural)**				
Urban Residence	0.743^**^	0.561 0.984	0.952	0.892 1.015
**Type of health provider (Ref. Public)**				
Private	2.019^***^	1.367 2.982	1.333^***^	1.239 1.433
**Type of healthcare (Ref. Outpatient)**				
Inpatient	23.528^***^	15.566 35.564	4.186^***^	3.744 4.680
Residual	0.0604^***^	0.033 0.110	0.217^***^	0.197 0.239

*** *p* < 0.01, ** *p* < 0.05

**Table A3:** Logistic regression model for determinants of CHE at 40% of household non-food expenditure

Variable	2010-11		2018-19	
	Odd Ratio	95% Conf. Interval	Odd Ratio	95% Conf. Interval
**Wealth status (Ref. Poorest)**				
Poorer	0.431^***^	0.319 0.582	0.716^***^	0.622 0.825
Middle	0.344^***^	0.249 0.474	0.575^***^	0.492 0.672
Richer	0.158^***^	0.104 0.239	0.563^***^	0.477 0.664
Richest	0.070^***^	0.040 0.121	0.402^***^	0.333 0.485
**Gender of household head (Ref. Male)**				
Female	1.096	0.528 2.275	0.846	0.652 1.098
**Age of household head (Ref. 16-30 years)**				
31–50 years	1.105	0.720 1.695	0.797^***^	0.686 0.926
51–60 years	1.27	0.726 2.242	0.915	0.757 1.107
60 above	0.917	0.445 1.891	1.407^***^	1.194 1.659
**Head’s education (Ref. Never attended school)**				
Attended school	1.098	0.826 1.461	0.94	0.854 1.04
**Head’s employment (Ref. Unemployed)**				
Employed	0.892	0.699 1.138	0.757^***^	0.676 0.849
**Household size (Ref. 1–3)**				
4–6	1.025	0.712 1.476	0.831^***^	0.734 0.939
7	0.999	0.699 1.427	1.034	0.894 1.195
**Place of residence (Ref. Rural)**				
Urban	0.739^**^	0.570 0.957	0.678^***^	0.604 0.762
**Type of health provider (Ref. Public)**				
Private	2.314^***^	1.589 3.369	1.412^***^	1.249 1.597
**Type of healthcare (Ref. Outpatient)**				
Inpatient	21.27^***^	14.217 31.824	6.243^***^	5.406 7.209
Residual	0.076	0.043 0.134	0.068^***^	0.058 0.080

*** *p* < 0.01, ** *p* < 0.05

## Data Availability

“This study is based on Household Integrated Economic Survey (HIES) 2010-11 and 2018-19 and National Health Accounts (NHA). HIES is available in public domain https://www.pbs.gov.pk/content/microdata whereas NHA data can be obtain against the payment from the Pakistan bureau of statistics.”
